# Ethics and *Total Worker Health^®^*: Constructs for Ethical Decision-Making and Competencies for Professional Practice

**DOI:** 10.3390/ijerph181910030

**Published:** 2021-09-24

**Authors:** Bonnie Rogers, Anita L. Schill

**Affiliations:** 1 National Institute for Occupational Saety and Health, Washington, DC 20201, USA; anita.l.schill@gmail.com; 2 Gillings School of Global Public Health, University of North Carolina, Chapel Hill, NC 27544, USA

**Keywords:** total worker health, professional competencies, ethical decision-making

## Abstract

Work has become increasingly technologically driven and fast paced, with long work hours, new/emerging hazards, and rising health care costs. Threats to worker safety, health, and well-being including non-traditional work arrangements and practices, precarious work, uncertain hazardous exposures, and work organization issues, such as heavy workloads, design of work, uneven work hours, and difficult interpersonal relationships among workers and managers are apparent. Furthermore, the relationship between personal health risk factors and workplace risks and exposures has drawn increased attention and concern. As employer economic pressures continue to build, it is anticipated that ethical dilemmas for practitioners will become increasingly complex. A review of relevant *Total Worker Health^®^* (TWH) literature, related ethical constructs and competencies, an examination of codes of ethics for occupational safety and health and health promotion/education disciplines was conducted. A case study for TWH utilizing an ethical decision-making model for the analysis of key ethical issues and solutions was completed. TWH approaches to protecting safety, promoting health, and advancing well-being are increasingly being adopted. These approaches can reveal ethical dilemmas, and ethical constructs are needed to guide decision-making. A core set of proposed ethical competencies for TWH professionals are identified as a transdisciplinary framework to support workplace ethical culture.

## 1. Introduction

There have been dramatic changes in the nature of work over the recent decades. Work has become increasingly technologically driven and fast-paced, with goods production and service provision more likely to span cycles of twenty-four hours a day, seven days a week. Unsafe work conditions characterized by legacy risks and hazards continue along with the introduction of new work environments that present issues not yet fully understood. As the future of work continues to evolve, it is anticipated these environments will bring new threats to the workplace including those associated with non-traditional work arrangements and practices, precarious work, and organizational design issues, such as long work hours, heavy workloads, and difficult interpersonal relationships among workers and managers, changes to the technologies associated with work, and changing workforce characteristics [1].

Furthermore, the relationship between personal health risk factors and workplace risks and exposures has drawn increasing attention [2,3]. As health care costs continue to rise, issues related to cost containment, such as disability management, have sometimes created unintended conflicts between employers and workers, often putting occupational safety and health professionals in the difficult position of choosing loyalty to their employer or advocating for affected workers. As economic pressures for employers continue to build and work environments continue to change, it is anticipated that ethical dilemmas for practitioners will become increasingly complex, requiring competent ethical decision-making strategies to address and resolve these issues.

The National Institute for Occupational Safety and Health (NIOSH) *Total Worker Health^®^* (TWH) approach to worker safety, health, and well-being is a comprehensive program that may provide guidance as professionals seek to address these concerns. According to NIOSH, TWH is defined as policies, programs, and practices that integrate protection from work-related safety and health hazards with promotion of injury and illness prevention efforts to advance worker well-being [4,5]. Traditional occupational safety and health protection programs have primarily concentrated on ensuring that work is safe and that workers are protected from the harms that directly arise from work. Building on this foundation, TWH supports a thorough understanding of factors that contribute to worker safety, health, and well-being, including hazards that arise from work and those that develop outside of work [6]. This approach recognizes hazards and exposures, such as those from biologic, chemical, enviromechanical, physical, and psychosocial agents, as well as workplace issues not previously thought to impact worker health, such as hours of work, access to paid leave, healthy leadership, and meaningful work. Scientific evidence now supports what many occupational safety and health professionals, as well as workers themselves, have long suspected—risk factors in the workplace can contribute to health problems previously considered unrelated to work, such as sleep disorders, obesity, musculoskeletal disorders, and cardiovascular disease [6,7]. As the artificial boundaries between work and home life continue to erode, future challenges related to worker safety, health, and well-being will become more intertwined with personal choice and, thus, create a need for ever-more intricate ethical decision-making approaches.

The purposes of this paper are twofold: to focus on ethical constructs for decision-making by occupational safety and health (OSH) professionals in organizations that have adopted TWH approaches to worker safety, health, and well-being, and to propose ethical competencies for practitioners who practice TWH approaches. As the practice of occupational safety and health expands beyond the traditional responsibilities for safety and health protection and worksite health promotion programs move beyond a primary focus on behavior change, professional practice will increasingly engage a TWH approach. This includes, for many, moving into uncharted territory for finding the balance between employer interests and worker well-being. Focused ethical decision-making in total worker health is essential as this practice grows.

## 2. Ethics

Ethics is a generic term that covers several different ways of examining, understanding, and applying moral principles that guide behavior and actions to address specific problems or dilemmas. Ethics are the standards of right and wrong that guide behavior. An ethical framework or code provides guidance for how people reason, problem-solve, and respond [8]. Ethics connotes a dynamic process of discussion and debate among individuals involved in the decision-making process. An ethical approach requires multidisciplinary cooperation and collaborative participation to arrive at a course of action infused with trust and integrity.

The ethical decision-making process itself may turn out to be more important than its ultimate outcome. That is, how one transparently arrives at decisions shows respect for and fosters trust in the process. Using ethical theories and principles as foundational, ethically derived policies that achieve the greatest benefit (with minimal or no harm) provide concrete guidance for moving forward during challenging circumstances. When confronted with such challenges, occupational safety and health professionals have a responsibility to use independent judgment based on scientific knowledge and technical competence, guided by their professional code of ethics, while avoiding real or perceived conflicts of interest. Ethical competence in applying these principles will support a total worker health ethical culture.

## 3. Ethical Principles

Basic ethical principles provide guidance for all occupational safety and health professionals to protect the rights of workers and support an ethical culture for safety, health, and well-being in the workplace. There are four basic principles: autonomy, nonmaleficence, beneficence, and justice. However, these principles are not always discrete. They may overlap and sometimes they may conflict with each other. It may be more helpful to consider these principles as a continuum for professional guidance.

The ethical principle of autonomy is a form of personal liberty concerned with the right to self-determination; it respects the individual’s right to make informed decisions [8]. Autonomy necessitates that personal values and goals be considered in major decisions affecting individual welfare and negates occupational safety and health professionals or others making decisions for a worker without autonomous input and consent. Autonomy encompasses the idea that everyone has the right to make his or her own choices in life. The principle of autonomy is especially predominant in Western cultures.

When applied to the workplace, complete and full disclosure regarding potentially harmful work is essential for informed decision-making and self-determination. Such disclosure includes information regarding work-related risks, known hazard or exposure interactions with personal health conditions, the right to know about potentially hazardous substances, and the pros and cons of accepting hazard pay for dangerous work. Inherent in this disclosure would be information about measures the employer is taking to protect workers and what decisions workers can make to protect themselves from harm.

In addition, when applied to worker participation in health screenings or health promotion programs, full disclosure is indicated to protect individual autonomy. Individual decision-making is best reflected through informed consent. Coercion and paternalism by management and health professionals regarding employee participation in company-sponsored programs, often tied to cost containment or discrimination against “high risk” workers, violate the principle of autonomy. Each individual worker should have the right to accept or refuse participation in health screening programs and medical treatment for health conditions. Health information and medical records generated by health care practitioners are private and confidential records and are to be protected from unauthorized access, unless otherwise required by law.

Employee autonomy must be safeguarded and of utmost concern is confidentiality and privacy of health information which can be at serious risk. Health information should be shared only with employee informed consent beforehand or when legally required to do so. The employee needs to trust that occupational health care providers will protect health information. Disclosure of personal health information without an employee’s prior consent can undermine that trust. Employees may decline to participate in voluntary worksite programs or fail to disclose relevant health information accurately and truthfully to health care providers if they believe the employer will use the information against them in an embarrassing or discriminatory manner.

It is recognized that employers do have legitimate needs for certain types of employee health information, such as health hazard surveillance data. However, the provision of aggregate data to management related to the health effects of work-related exposures should be adequate. Management can also be informed about an individual’s fitness to work or illness/injury-related work absences without disclosure of diagnosis or other personal health information unrelated to the employee’s ability to perform the job. There is no need for management to “second guess” the health professional’s expert judgment about health/medical conditions. The issue of confidentiality and privacy of employee health information is an ethical tenet that should not be violated. No real benefit can be reaped by failure to respect employees’ privacy through unwarranted access to personal health information that has no relevance to fitness to work.

Occupational safety and health practitioners are ethically bound to prevent practices related to confidential health information which are ethically unsound, such as misuse or abuse of occupational health data, ignoring informed consent, concealing or withholding findings, and inadequate protection of worker health records, or data, whether in physical paper or electronic format. Only when individual anonymity is assured can aggregate group health data of workers be disclosed to management and workers’ representatives with the goal of protecting and promoting worker health and safety. Unless required by law, personal health information that is not relevant to the protection, maintenance, or promotion of workers’ health in relation to work or to the overall health of the workforce must be protected as confidential.

Nonmaleficence is often referred to as the “no harm principle” [8] and it is inherent in professional standards, licensure, and codes of ethics. Within the construct of this ethical principle, occupational safety and health professionals have an obligation to protect workers from the risk of harm. Any potential risk of an intervention to protect safety and health should never outweigh the benefits of the intervention.

For example, a pregnant employee should not be exposed to known or potential teratogens, such as antineoplastic agents or prolonged standing, which may compromise the health of the woman or unborn baby. Another example of the no-harm principle is to avoid the placement of a worker with a known respiratory disease in work that would create or aggravate respiratory distress, including exposure to passive cigarette smoke. A third example relates to employing a young worker in a battery manufacturing company with the potential for lead exposure from welding operations. Although this worker may not be a welder, there will likely be exposure to lead as work takes place in close proximity to welding activities and personal protective equipment, including respiratory protection, is not provided.

Another consideration of nonmaleficence in the workplace is the harm caused by one worker to other co-workers, such as a forklift driver who is actively misusing a substance known to impair driving and judgment, or a worker who engages in bullying behaviors. In the case of the forklift driver, a breach of confidentiality that may violate the principle of autonomy may be necessary to protect the co-workers. Fitness for duty assessments provides an avenue to assist impaired workers, making sure they receive adequate care and treatment, prior to resumption of work responsibilities. Ethical practice would direct health care professionals to advocate for the health of the worker, regardless of conflicting pressure applied by management.

Furthermore, the issue of workplace stress is significant and can be harmful to health and well-being [9]. This is another example of the “no harm” principle where stressful acts and situations must be mitigated or eliminated. Stress is not just emotion; it affects body systems and the ability to do work. Often stress is caused when workers feel little or no control over what they are doing, such as unfair assignments, heavy workloads, long work hours, unfulfilling jobs, dangerous working conditions, bullying, and violence. Unmanaged stress may cause workers to take unsafe shortcuts to produce more, become engaged in conflict, or assume unhealthy behaviors, such as smoking, drug misuse, or poor eating [9].

Beneficence, the third ethical principle, pertains to providing benefits, preventing or removing harm, and balancing benefits against risks and costs [8]. It is the principle that supports occupational safety and health professionals in their efforts to assist workers to achieve their maximum health potential. Interventions aimed at health protection, such as the implementation of the TWH hierarchy of controls [6], health screening for adverse work-related health effects, and surveillance programs exemplify beneficence. The implementation of health-promoting programs, such as smoking cessation, exercise, and good nutrition, also illustrates this principle. Additional examples of beneficence include:

- Conducting team walk-throughs to identify potential hazards and make recommendations for hazard abatement;
- Targeting potential causes of stress be they work (e.g., work overload) or non-work-related (e.g., lactation support) for risk reduction;
- Providing health screenings, such as preplacement and periodic examinations, that may identify early disease or disease interactive agents (e.g., noise exposure and presbycusis);
- Providing protective immunizations (e.g., influenza vaccine; Sars-CoV-2 vaccine);
- Placement of engineering controls or development of a back-injury prevention program for workers with previous back injury;
- Assuring a worker with a disability that they will receive the best possible disability health care, through providing qualified health care providers;
- Providing adequate and continuous training so workers know how to do the job and are aware of potential hazards and their rights and responsibilities.

The TWH approach seeks to both protect workers (nonmaleficence) and promote their well-being (beneficence).

The fourth ethical principle, justice, is directed toward treating workers equally, without discrimination, and distributing benefits, risks, and costs [8]. The principle of justice is embodied in the Americans with Disability Act of 1990 [10] which includes equal opportunity regarding job availability and promotion for individuals with disabilities and those with preexisting conditions. It aims to prevent discrimination against individuals who are capable of performing all job-required job tasks despite the presence of a health condition.

The ethical principle of justice includes the concept of equity. There are numerous definitions of health equity. In general, they incorporate the goal of achieving the highest level of health and well-being for all people, regardless of social and economic status, by eliminating disparities, including disparities related to labor and employment conditions [11]. Health equity is consistent with the TWH approach of protecting all workers from safety and health hazards and advancing the well-being of all workers. Selecting minority or disadvantaged groups of workers based on a common defining characteristic to perform unpleasant or hazardous jobs violates the principle of justice and the goal of health equity. All workers should be treated and respected equally.

## 4. Ethical Decision-Making

The ethical principles provide guidance for occupational safety and health professionals as they consider the best course of action to take to address issues related to worker safety, health, and well-being. The challenge presented by the TWH approach is the broad assortment of conditions related to work that fall under the TWH umbrella. Thus, practitioners of TWH may be challenged in directions where there is little established guidance. When confronted with ethical issues involving right and wrong, practitioners will naturally rely on their personal and professional values, professional codes of ethics, legal obligations, and the moral code of behavior to which they adhere [12]. However, sometimes a decision-making challenge turns into a conflict or an ethical dilemma.

Ethical dilemmas are difficult problems that have no perfect solutions. They have been defined as “a decision-making problem between two possible moral imperatives, neither of which is unambiguously acceptable or [sic] preferable” [13]. Allen [12] contends that three conditions must be met for a problem to become an ethical dilemma: a choice must be made, there are different possible solutions, and no matter which solution is selected, an ethical principle is violated.

Curtain [14] describes that as various ethical dilemmas are analyzed there will be some situations in which one value conflicts with another, other situations in which duties conflict, or still others where duties conflict with the desired outcome. It may be that what the practitioner wants to do conflicts with what the practitioner thinks ought to be done [15]. The application of general ethical principles may help clarify the problem and guide decision-making.

For example, depending on the work being performed, workers can be exposed to hazardous chemicals or substances and they may not have an adequate understanding of the risks and consequences of their exposure. The legal basis for notifying workers about exposure to toxic substances is clearly embodied in the Occupational Safety and Health Act [16], the Toxic Substances Control Act of 1976 [17], and the Hazard Communication Standard of 1983 [18]. Underlying these legal requirements are the ethical principles of autonomy and nonmaleficence.

Precepts related to “right to know” are embodied in the principle of autonomy and include access to information, right to self-determination, informed consent, and participation in decision-making. Workers have the right to know about hazardous substance exposure and make informed decisions about their options to limit exposure. They also have the right to develop their own course of action by engaging with the employer to better design and implement safety and health programs, procedures, and equipment to mitigate or eliminate the exposure risk.

Precepts related to “no harm” are embodied in the principle of nonmaleficence. As related to toxic agent exposures, harm can include both short-term and latent health effects on personal health, reproductive toxicity, concern about mental and physical integrity of offspring, fear and anxiety about health effects, and employer costs and benefits [19]. However, regardless of the legal requirements and specific standards designed to protect worker health and prevent harm, workers continue to be exposed to toxic substances and conditions in the workplace that are harmful to them and, potentially, to their families.

Also of importance is the precautionary principle that indicates that when an activity raises a reasonable suspicion of causing harm to human health, though there is no scientific evidence, precautionary measures should be taken just as if proof of its damage actually existed [20]. In the workplace, employers should take every precaution to protect workers from hazards and the precautionary principle should apply in situations where there is no definitive scientific evidence regarding the potential risk posed by a hazard and controls applied related to the hazard [21]. It asserts that when there is suspected harm and the scientific evidence is inconclusive, the prescribed course is precautionary action. The precautionary principle is most powerful when it serves as a guide to making wiser decisions in the face of uncertainty [22]. Protection of workers from uncertain hazards and risks requires the precautionary principle which is considered good practice.

## 5. Case Study

**Figure 1:**
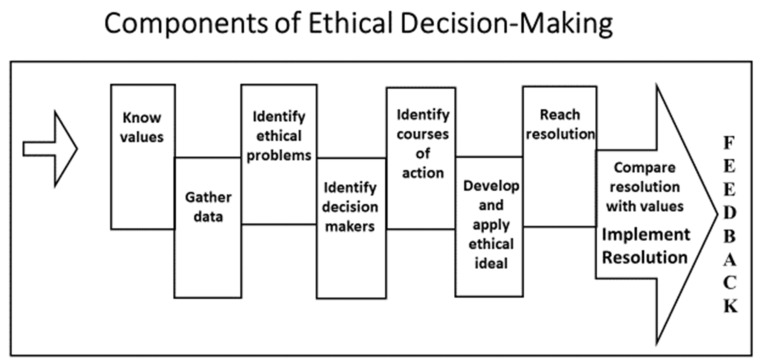
Components of Ethical Decision-Making.

A framework or model for ethical analysis [23,24] may be beneficial to work through ethical dilemmas and support the formulation of ethical decisions (Figure 1). The case study approach will be used to demonstrate the application of a ten-step, ethical decision-making model. The ten steps are:

1. Know values
2. Gather data
3. Identify ethical problems
4. Identify decision-makers
5. Identify courses of action
6. Develop and apply the ethical ideal
7. Reach a resolution
8. Compare the resolution with values
9. Implement the resolution
10. Feedback

## 6. Case Presentation

You are an occupational safety and health professional in the corporate offices of a long-term care facility with multiple locations in the Midwest. One year ago, you successfully convinced your boss and other key corporate executives of the benefits of implementing a TWH program for all workers in the local residential facilities. You were authorized to begin the implementation of a TWH program with a mandate to improve worker satisfaction and help with staff retention. Staff members are primarily nurses’ aides, but also include nurses, dieticians, rehabilitation therapy staff, recreational therapists, social services staff, housekeeping staff and maintenance staff. You are in the initial stages of TWH program implementation.

This week you received notification that the corporation recently changed health care insurance companies and a new premium structure has been negotiated. Subsequently, you received a phone call from your boss, the corporate vice president of risk management (VP). During the call, the VP advised you of the high health insurance costs related to the number of facility staff who are overweight or obese. In fact, that is why the previous insurance company did not offer a renewal when the term of the prior policy expired.

The VP directed you to implement a mandatory weight loss program for workers who are overweight or obese and has suggested that successful implementation will lead to a substantial bonus for you and perhaps even a promotion. The VP has determined that worker weight loss will lower health insurance costs and reduce workers’ compensation claims due to injuries resulting from lifting and assisting residents with limited mobility. You subsequently find out that staff members who do not achieve a “normal” weight within the next six months will be penalized with higher health insurance premiums.

As an occupational safety and health professional and TWH practitioner, you know that a weight loss program alone may be insufficient to help staff members achieve a weight in the normal range [25], especially in the proposed six-month period. You believe a comprehensive approach is needed to assist staff members who are overweight or obese. You are aware that some facilities are understaffed, resulting in nurses’ aides and other staff often having to work double shifts. You are also aware that staffing shortages result in highly stressful working environments and you worry that care for the residents is compromised, which creates more stress for the staff. Your previous requests for mechanical lift devices, while supported by the nursing staff, have been rejected due to cost, training needs, and perceptions that residents will ultimately reject them. What do you do?

## 7. Ethical Analysis

### 7.1. Know Values

Begin the process of sorting through the issues by identifying the relevant values. Values identification fosters clarification of and reflection on applicable belief systems, including those of the individual doing the analysis. It creates an understanding of self-perspective as a reasoned approach to ethical problem analysis is developed.

In addition to the values held by the individual leading the ethical analysis, it is also critical to consider corporate values, perhaps as put forth in formal vision, mission, and value statements. Other values that are important to identify upfront include those of the involved workers, worker representatives, managers, corporate representatives, and any others who are contributing to the discussions and problem-solving. These values and belief systems should be stated clearly upfront and used to guide the ethical analysis process.

In the case presentation, evident values and beliefs include:

- Corporate executives value a TWH program, specifically to improve worker satisfaction and retention.
- The VP values healthier workers but focuses on the attainment of a healthy weight.
- The corporation values workers by offering health care insurance but believes premiums could be lower if workers were healthier. Lower health insurance premiums would improve the financial health of the corporation and could save money for the workers themselves.
- The TWH practitioner believes a comprehensive approach is needed to address worker health issues, including weight, stress, fatigue, and lifting injuries.
- The TWH practitioner has been offered monetary and prestige incentives to implement the mandatory weight loss program.
- A TWH program is of value to some workers.
- Informed consent for workers and confidentiality of health information is valued by the OSH/TWH professional and workers but their value to corporate executives is uncertain given the directive for a mandatory program.

### 7.2. Gather Data

In order to proceed with the ethical decision-making process, the fullest possible range of information and data pertinent to the problem should be collected. Sources for this data collection activity should include all of those affected by the problem and potential solutions. Additionally, corporate financial data may also create perspective for eventual problem-solving. Examples of information and data that may aid discussions include:

- Activities undertaken to date for TWH program implementation including the workers’ input who have been involved in program development.
- Demographic data of workforce.
- Aggregated statistics related to the prevalence of workers who are overweight and obese, and the associated insurance costs as compared with workers whose weight is in the normal range.
- Aggregated statistics on workers’ compensation for worker injuries related to unsafe resident handling procedures.
- Potential burden of health insurance penalties for those who do not achieve the desired weight loss.
- Staffing to resident ratios; current and recent trends.
- Use of mandatory overtime, including double shifts and perceived stress levels among the workers.
- Issues and formal complaints regarding compromised quality of care for residents.
- Costs associated with mechanical lift devices including equipment and training.
- Types of meals offered at the facilities.
- How the confidentiality of health data is managed.

### 7.3. Identify Ethical Problems

Recall that ethical dilemmas are problems that elude perfect solutions. While there may be different courses of action, choosing one action may support one ethical principle while violating or compromising another ethical principle. However, the ethical principles of autonomy, nonmaleficence, beneficence, and justice, can be used as guides for ethical decision-making and to identify ethical intrusions into the rights of workers. Examples of how these principles might potentially be violated in the case study include:

- Autonomy—Coercion and paternalism by management for mandatory worker participation in a weight loss program; lack of worker informed consent; pressure from management for OSH/TWH professional to conduct a mandatory weight loss program, with a potential conflict of interest for bonus pay/incentives, which could be in conflict with the professional code of ethics and also considered professionally offensive.
- Nonmaleficence—Staff shortages resulting in mandatory overtime, including double shifts for workers creating stress, fatigue, and potential practice errors; exposure to continued unsafe lifting practices of residents create a further risk of harm; failure to achieve weight loss creating worker poor self-image and concern for continued employment.
- Beneficence—Purchase of mechanical device equipment rejected by management because of cost; company meals provided are mostly fatty or fried food.
- Justice—Health insurance penalties for those who do not or who are unable to lose the required amount of weight in the defined period discriminate against this worker group; a monetary penalty may impact worker salary.

### 7.4. Identify Decision-Makers

Failure to deal with this component of the ethical decision-making process could result in the wrong people, making the wrong decisions, for the wrong reasons. Since the TWH approach to worker safety, health, and well-being is a transdisciplinary effort, many people may be involved in program development. While the number of decision-makers may be large, making the solution-generating process a bit unwieldy, a better outcome is achieved when those affected by the decisions are involved in the process.

In the case study, decision-makers who should be involved in the process include: workers from each department of the local residential facilities, worker representatives (if there are unionized facilities), facility managers, the VP and other corporate executives as appropriate, occupational safety and health/TWH professionals, and dietician. Consideration should also be given to including one or more residents to represent their local facility.

### 7.5. Identify Courses of Action

By definition, an ethical dilemma will have several potential courses of action, none of them perfect. In this case study, alternative courses of action may include:

- Ask a representative from each department of the local residential facilities to offer one or two suggestions for making the work environment safer and healthier and improving the health of staff members.
- Offer voluntary weight management program with incentives for weight loss, including new uniforms to celebrate weight loss achievements; eliminate health insurance penalties for workers who are unable to achieve the desired biometrics in the defined time.
- Develop a plan for using dietary staff and kitchens in residential facilities to provide healthier meal options for staff during their work shifts.
- Identify funding options (budget, grant, gift) for purchase/rental of mechanical lift devices for residents with limited mobility. Eliminate the need for staff bonuses.
- Focus efforts on becoming a community employer of choice to help eliminate staffing shortages (long-term).
- Refuse to implement weight loss program unless voluntary for workers, without penalty, and with informed consent and confidentiality of health information and seek higher chain of command for discussion. Indicate that this would be a violation of the professional code of ethics.

### 7.6. Develop and Apply Ethical Ideal

At this stage in the ethical decision-making process, each of the ethical principles—autonomy, nonmaleficence, beneficence, and justice—should be considered in relation to potential courses of action under discussion. For example, in the potential courses of action identified above, involving affected workers in the decision-making process supports the principle of autonomy. The principle of nonmaleficence is evident in efforts to eliminate staffing shortages, provide mechanical lifts, and eliminate the potential financial hardship of buying new uniforms when weight loss is successful. Providing healthier or lower-cost meal options through existing dietary staff and kitchen facilities supports the principle of beneficence. Eliminating the threat of health insurance penalties creates a more just work environment. Once each potential solution is screened for ethical soundness and modified as needed to align with the principles, problem resolution becomes more surefooted.

### 7.7. Reach Resolution

In all ethical decision-making, each potential course of action should be given serious consideration, especially for alignment with the ethical principles. Successful resolution options will, by necessity, be feasible, practical, affordable, and ethical. As a resolution becomes clearer, it may be helpful to create an ethical statement that addresses what can be done, by whom and under what conditions, rationale for the selected course of action, and feasibility of implementation.

In the case study, an example of an ethical statement might be:

It has been decided that a voluntary weight loss management program will be the most effective approach and will be implemented under the direction of the OSH/TWH professional. The decision for this voluntary program is that it will give workers a greater say in the decision-making about their personal health (autonomy) rather than being coerced into forced participation. Workers will be able to set personal achievement goals including weight loss within the desired timeframe. They will get support, direction, and encouragement from health professionals and may feel significant accomplishment in meeting their goals (beneficence) with potentially less fear of failure to achieve (nonmaleficence) and encouraged to continue with weight management once the program is completed. Health information obtained will be kept strictly confidential by the health professional (autonomy) and only aggregate data will be shared with management. There will be no penalty for non-participation (justice). Incentives will be offered with worker input and with budgetary considerations from the VP of human resources. Other non-participant workers may want to engage once they see the value and success of the program (beneficence).

### 7.8. Compare Resolution with Values, Implement, and Provide Feedback

Once a resolution has been stated, it should be checked for alignment with values. For example, the above ethical statement aligns with this value statement: This corporation values its workers and, therefore, is committed to improving their safety, health, and well-being (beneficence), including opportunities for workers to participate in decisions that affect them (autonomy). As an application of the resolution proceeds, progress can be assessed by checking in with those responsible for implementation and those who are affected by the resolution. Assessment of the implementation might include answers to questions such as: what outcomes are evident to the affected workers, have corporate objectives been achieved, have the intended benefits been realized by the intended workers, are modifications to the resolution needed, and were the costs associated with resolution acceptable. Seeking this information can be accomplished through confidential interviews, observations, group meetings, and appropriate discussions with management. The ideal resolution is one that affords the most benefit with the least amount of harm while treating all affected individuals fairly and creating options for participation.

## 8. Professional Codes of Ethics

Health professionals including those in occupational safety and health, health education, and health promotion will find guidance for ethical decision-making in their respective codes of ethics. Professional associations develop codes of ethics to serve as a framework for guiding professional practice and outlining accountability to the consumer. These codes embody collective philosophies about the values and beliefs for professional practice. In addition to the principles of ethics previously discussed, members of the corresponding profession should use professional codes to guide ethical decision-making.

The American Association of Occupational Health Nurses (AAOHN) and the American College of Occupational and Environmental Medicine (ACOEM) both have codes of ethics that closely track ethical principles [26,27]. At least one of the principles of autonomy, nonmaleficence, beneficence, and justice is embedded in each of the ethical statements that comprise the AAOHN and ACOEM codes. Of the ethical codes for occupational safety and health professionals, the AAOHN and ACOEM codes are unique in that they specifically address issues related to the confidentiality of medical and health information.

Industrial hygienists are served by a code of ethics issued jointly by the American Industrial Hygiene Association (AIHA) and the American Conference of Governmental Industrial Hygienists [28]. For those industrial hygienists who are certified, a certified associate, or a candidate for certification, the American Board of Industrial Hygienists (ABIH) has a code of ethics that closely tracks the AIHA code [29]. While these codes address issues focused on the industrial hygiene profession, the ethical principles are evident in several of the ethical statements. Of note, as related to the case study, one statement addresses refraining from accepting payments that are intended to influence professional judgment. The American Society of Safety Professionals (ASSP) also has a code of professional conduct [30]. Again, the ethical principles are reflected in several of the statements created to guide the practice of safety professionals.

The Society for Public Health Education follows the Health Education Code of Ethics developed by the Coalition of National Health Education Organizations [31]. Similar to the occupational safety and health codes, this is grounded in fundamental ethical principles of honesty, autonomy, beneficence, respect, and justice. These professionals advocate and encourage actions and social policies that promote maximal health benefits and the elimination or minimization of preventable risks and health inequities for all affected parties. They are ethically bound to respect the privacy, confidentiality, and dignity of individuals and organizations.

However, there may be a gap between the code and real-life practice, confusion between the various codes, and the legal standing of these codes is uncertain [13]. In response, the International Commission on Occupational Health (ICOH) [32] has developed the world standard ethical code for occupational health professionals, covering all the disciplines of professionals whose practice relates to work and the workplace. Some countries have adopted the ICOH code of ethics in their legislation related to occupational health professionals [13].

## 9. Ethical Competencies in Total Worker Health

Competencies for occupational safety and health practitioners were originally defined in 1950 by the International Labour Organization (ILO) and World Health Organization (WHO). They were updated by the ILO/WHO Joint Committee on Occupational Health in 1995 with the following text:

Occupational health should aim at: the promotion and maintenance of the highest degree of physical, mental and social well-being of workers in all occupations; the prevention amongst workers of departures from health caused by their working conditions; the protection of workers in their employment from risks resulting from factors adverse to health; the placing and maintenance of the worker in an occupational environment adapted to his physiological and psychological capabilities; and, to summarize, the adaptation of work to man and of each man to his job. The main focus in occupational health is on three different objectives: (i) the maintenance and promotion of workers’ health and working capacity; (ii) the improvement of working environment and work to become conducive to safety and health; and (iii) development of work organizations and working cultures in a direction which supports health and safety at work and in doing so also promotes a positive social climate and smooth operation and may enhance productivity of the undertakings. The concept of working culture is intended in this context to mean a reflection of the essential value systems adopted by the undertaking concerned. Such a culture is reflected in practice in the managerial systems, personnel policy, principles for participation, training policies and quality management of the undertaking [33].

A wide range of disciplines work as occupational safety and health professionals including occupational health physicians, occupational health nurses, industrial hygienists, safety specialists, ergonomists, health educators, health promotion specialists, occupational health psychologists, occupational health rehabilitation therapists, employee assistance program therapists, and others. As the TWH approach continues to be adopted in workplaces, it will be important for all disciplines to support a shared set of values and understand each other’s responsibilities and professional standards as they relate to ethical decision-making. The competence of these practitioners should be mobilized within the framework of a transdisciplinary team approach to provide leadership for the improvement of the working environment and working conditions, including the design of work with health outcomes in mind, as well as worker safety, health, and well-being [34,35,36]. Aligning the concept of TWH with an understanding of ethical principles, a proposed set of ethical competencies is presented in Table 1.

## 10. Conclusions

Due to their unique practice settings, occupational safety and health professionals have always faced the potential of navigating situations where the interests of multiple parties conflict, thereby creating decision-making challenges. As more occupational safety and health practitioners adopt TWH approaches to worker safety, health, and well-being, it is anticipated that such conflicts will increase and become even more difficult to resolve. Further complicating the decision-making environment for occupational safety and health professionals will be the evolving nature of work environments, the addition of existing and yet-to-be-discovered technologies to accomplish work and an increasingly diverse workforce. Fortunately, there are accessible tools to aid practitioners, including the basic ethical principles of biomedicine, professional codes of ethics, and the universal ethical code developed by ICOH.

An additional tool to aid occupational safety and health professionals is presented here as a model that delineates the components of ethical decision-making. By working through each component in the model, as demonstrated in the case study, practitioners will have a systematic approach to problem-solving for ethical dilemmas. Using a systematic approach such as this will guide decision-makers as they balance multiple perspectives and an evolving work environment landscape to achieve the greatest benefit with minimal or no harm and fairness to all involved.

A proposed set of ethical competencies for TWH practitioners is presented to stimulate thought about emerging requirements for occupational safety and health professionals who adopt TWH approaches. Educators and continuing education providers will find opportunities in these proposed competencies for expanding offerings to those whose practice includes TWH approaches to worker safety, health, and well-being. These competencies will, by necessity, evolve as the field matures.

The next steps for the intersection of ethics and TWH may be considered from several perspectives. First, research focused on this nexus would provide evidence-based support for TWH practitioners as they navigate through difficult issues. Second, the creation of standards of practice that incorporate the proposed ethical competencies for TWH practitioners would create a foundation of support for the implementation of TWH approaches in diverse practice settings. Professional organizations that support TWH practitioners should consider amending existing practice standards to specifically address potential ethical challenges posed by TWH approaches. Third, the process of policy development in organizations committed to protecting worker safety, promoting worker health, and advancing worker well-being should incorporate components of ethical decision-making and considerations of health equity to achieve total worker health for each and every worker.

## Figures and Tables

**Table 1:** Ethical Competencies in Total Worker Health.

1. Provide non-discriminatory care or service, based on the principle of equity, to protect health related to work.
2. Practice with professional independence, based on knowledge, scientific evidence, good practice, and the highest professional standards, when making decisions related to the protection and promotion of worker health and safety.
3. Emphasize primary prevention, defined in terms of worksite assessment, policies, design, technology, and practice, to determine if workers can safely complete the required job tasks.
4. Protect confidentiality of individual employee health and medical data and prevent misuse of these data to respect human dignity, protect privacy, and enhance the acceptability and effectiveness of occupational health practice for physicians and nurses.
5. Use validated methods for risk assessment, monitoring, and health promotion, propose effective preventive measures, assess effectiveness of implementation, and be proactive in improving health and safety for workers based on professional codes for competency and ethics.
6. Recognize danger signs for violent or suicidal employees, take appropriate action, make referral, and follow up as appropriate.
7. Provide sound and honest advice to employers about legal, regulatory, and ethical responsibility in the field of occupational safety and health, and to workers for the protection and promotion of their health in relation to work.
8. Balance ethical principles with need to report hazards impacting others.
9. Understand the concepts of risk perception, acceptable risk, and risk communication.
10. Recognize prevention efforts and programs should be provided at both individualized and population-focused levels and be implemented with non-coerced, informed worker consent, including a discussion of potentially positive and negative consequences of participation in screening and health surveillance activities. Screening tests or unproven methods, which are not reliable, or methods that overly invasive should be avoided.
11. Provide guidance to workers and employers about optimal health and productivity programs and the value of health promotion programs, such as those for smoking cessation, physical activity, healthy eating, and fatigue prevention.
12. Provide multidisciplinary team support to the organization in creating and sustaining a culture of health at both system and individual levels while respecting the cultural and health beliefs of workers.
13. Remain knowledgeable about the work, work organization, and the work environment. Be well informed on current scientific and technical knowledge about occupational hazards from biological, chemical, enviromechanical, physical, and psychosocial agent exposures and the most efficient and effective means to eliminate or to minimize toxic agent exposure risk.
14. Conduct and participate in occupational health and safety program evaluations and audits to provide for continual improvement.
15. Contribute to the information for workers on occupational hazards to which they may be exposed in an objective and understandable manner, which does not conceal any fact, recognizes uncertainties of known or suspected hazards, and emphasizes preventive measures while addressing language barriers and cross-cultural differences.
16. Understand that determination of fitness for job duty must be based on a good knowledge of the job demands, essential job functions, the worksite, and assessment of the health of the worker.
17. Seek the participation of both employers and workers in the design and implementation of health education, health promotion, health screening, and public health programs.
18. Design and carry out research activities on a sound scientific basis within the context of ethical principles relevant to health and medical research work.

## Data Availability

No data reported.
